# Improved Survival of Leukemic Mice Treated with Sodium Caseinate in Combination with Daunorubicin without Toxicity

**DOI:** 10.1155/2021/6635650

**Published:** 2021-02-27

**Authors:** Itzen Aguiñiga-Sánchez, Frida Montserrat Meléndez-Ibarra, Edgar Ledesma-Martínez, Benny Weiss-Steider, Guadalupe Rosario Fajardo-Orduña, Rosalva Rangel-Corona, Sac-Nicte García-Gervasio, María Guadalupe Ramírez-Padilla, José Luis Lara-Castañeda, Edelmiro Santiago-Osorio

**Affiliations:** ^1^Hematopoiesis and Leukemia Laboratory, Research Unit on Cell Differentiation an Cancer, Faculty of High Studies Zaragoza, National Autonomous University of Mexico, 09230 Mexico City, Mexico; ^2^Department of Biomedical Sciences, School of Medicine, Faculty of High Studies Zaragoza, National Autonomous University of Mexico, 09230 Mexico City, Mexico; ^3^Cellular Oncology Laboratory, Research Unit on Cell Differentiation an Cancer, Faculty of High Studies Zaragoza, National Autonomous University of Mexico, 09230, Mexico City, Mexico

## Abstract

In recent years, low doses of chemotherapy have been resumed and explored for the treatment of acute myeloid leukemia. Thus, CPX-351, a dual-drug liposomal encapsulation of cytarabine and daunorubicin, was approved by the US Food and Drug Administration, to deliver a synergistic 5 : 1 molar drug ratio into leukemia cells to a greater extent than normal bone marrow cells and significantly enhance survival compared with conventional treatment in older and newly diagnosed AML patients, but overall survival rate remains low; therefore, the need for new therapeutic options continues. Sodium caseinate (SC), a salt of casein, the main milk protein, has cytotoxic effect in leukemia cell lines, but promotes proliferation of hematopoietic normal cells, while its administration in leukemic mice promotes survival for more than 40 days, but bone marrow surviving mice still harbour leukemic cells, but it is not known whether the combination with cytarabine or daunorubicin can improve survival without damaging normal hematopoietic cells. Here, it is shown that, *in vitro*, the combination of the IC_25_ of SC-cytarabine or SC-daunorubicin synergizes in the elimination of leukemic cells, with evident induction of apoptosis, while the proliferation of mononuclear cells of bone marrow is not affected. In leukemic mice, the combined administration of SC-daunorubicin or SC-cytarabine promotes the highest survival rate at 40 days; in addition, no autoproliferating cells were detected in the bone marrow of survivors of more than 60 days, evidence of eradication of leukemic cells, but only the bone marrow of mice treated with the SC-daunorubicin combination proliferated in the presence of interleukin-3, which shows that this combination is not toxic to normal bone marrow cells, thus emerging as a possible antileukemic agent.

## 1. Introduction

Notwithstanding decades of research devoted to understanding the clinical and biological features of acute myeloid leukemia (AML), a group of heterogeneous diseases characterized by a clonal disorder in the myeloid progenitors of the bone marrow [1, 2], long-term survival is reduced in a large number of patients and the treatments have not made a significant advance to increase life expectancy [3, 4]. In this sense, leukemia continues to be treated based on the combination chemotherapy scheme [5], divided into induction, consolidation, and maintenance therapy, in addition to resorting, when possible, to hematopoietic stem cell (HSC) transplantation [5, 6].

Generally, induction therapy, called the “intensive” or 7 + 3 regimen, consists of combining cytarabine with an anthracycline (such as daunorubicin or idarubicin). However, not all patients are candidates for this regimen [6] as daunorubicin is highly toxic and can damage cardiomyocytes [7]. Thus, in young patients who achieve remission, only 40% to 50% survive 5 years and patients older than 60 years or diagnosed with an unfavorable risk group barely survive (10% to 20%) [8, 9].

An alternative to improve survival outcomes versus standard care chemotherapy is liposomal encapsulation, which allows drugs to be released in a controlled way and to reach the leukemic target more easily. CPX-351, a liposomal encapsulation of cytarabine and daunorubicin at a synergistic ratio into leukemia cells to a greater extent than normal bone marrow cells, was approved as Vyxeos in the US and EU for adults with newly diagnosed therapy-related AML or AML with myelodysplasia-related changes, and improved outcomes were observed with CPX-351 vs. 7 + 3 irrespective of baseline BM blast percentage in older adults with newly diagnosed high-risk/sAML [10]; however, the survival ratio is still low and the possibility of long-term relapse-free survival is questionable [11]. For this reason, the search for new treatment regimens based on combinations of chemotherapy drugs at low doses and administration seems to be a viable option.

Sodium caseinate (SC), a salt derived from casein, the main milk protein, inhibits the proliferation of leukemic cell lines such as J774, P388, and WEHI-3 [12]. It also promotes the production of hematopoietic regulators [13, 14] and the survival of leukemic mice and attenuates the manifestations of the disease [15, 16]. However, although it promotes survival, it is not capable of completely eradicating leukemic cells in the bone marrow (BM) [16], but it is not known whether this is achieved by combining it with cytarabine or daunorubicin. In this work, the effect of the combinations of CS with cytarabine or daunorubicin on the WEHI-3 cell line and bone marrow mononuclear cells (BMMNCs) from healthy mice, survival in leukemic mice, and the prevalence of leukemia are analyzed.

## 2. Materials and Methods

### 2.1. In Vitro Studies

#### 2.1.1. Cell Line and Culture Conditions

The WEHI-3 (mouse myelomonocytic leukemia) cell line was obtained from the ATCC (American Type Culture Collection, Virginia, USA). The cells were cultured in hydrophobic surface Petri dishes (Sarstedt AG & Co., Nümbrecht, Germany) with Iscove's modified Dulbecco's medium (IMDM) (Gibco-BRL, Carlsbad, CA, USA) supplemented with 10% fetal bovine serum (FBS) (Gibco-BRL, Carlsbad, CA, USA), 100 units/ml penicillin, and 100 *μ*g/ml streptomycin (Sigma-Aldrich, St. Louis, MO, USA). The cells were maintained in a humidified atmosphere with 5% CO_2_ at 37°C (Thermo Fisher Scientific Inc., Massachusetts, USA), and the culture medium was changed every 48 h.

#### 2.1.2. Bone Marrow Mononuclear Cell Collection

Clinically healthy mice of the Balb/c strain were euthanized by cervical dislocation. The femurs were obtained under sterile conditions and the total bone marrow cells were collected. Mononuclear cells (BMMNCs) were isolated from total cells via gradient separation with Ficoll-Histopaque (Sigma-Aldrich, St. Louis, MO, USA) at a density of 1.077 g/mL, and they were washed twice with phosphate-buffered saline (PBS). MNCs were cultured for 120 h in IMDM supplemented with 15% (v/v) FBS, 5% (v/v) horse serum (Gibco-BRL, Carlsbad, CA, USA) and 5 ng/mL recombinant mouse interleukin-3 (rmIL-3; R&D System, Minneapolis, MN, USA) or PBS. The cells were cultured in a humidified atmosphere with 5% CO_2_ at 37°C for a maximum duration of 120 h.

#### 2.1.3. Cell Proliferation Assays

For the evaluation of the antiproliferative activity of the drugs, WEHI-3 line and normal BMMNC were used, culturing them at a density of 5 × 10^3^ and 1 × 10^5^ cells mL^−1^ in culture medium, respectively, under the conditions described above. At time zero, the cells were stimulated with different concentrations of SC (0.25, 0.5, 1, 2, and 4 mg/mL) (Spectrum, New Brunswick, NJ), daunorubicin (0.6, 1.25, 2.5, 5, 10, 20, and 40 ng/mL) (Pfizer, New York, USA), or cytarabine (0.6, 1.25, 2.5, 5, 10, 20, and 40 *μ*g/mL) (Pfizer, New York, USA), and they were stimulated with PBS as a vehicle or cero. The culture was maintained for 72 h for the WEHI-3 cell line and 120 h for the mononucleated cells from normal mouse bone marrow. After the incubation time, cell proliferation was evaluated by the crystal violet technique modified by Kueng (1989) and originally proposed by Gillies et al., 1986. After 72 h in culture, the cells were fixed with 1% glutaraldehyde (Sigma-Aldrich, St. Louis, MO, USA) for 1 h, and a crystal violet (Sigma-Aldrich, St. Louis, MO, USA) dye solution was added to label the nuclei [17, 18] and quantify the cell number using a plate reader at 590 nm (Multiskan GO; Thermo Fisher Scientific Inc., Massachusetts, USA) [19]. The data were plotted, and the IC_50_ was obtained using a linear regression equation. The data obtained were reported in percentage of proliferation. After this, the IC_25_ of each drug was calculated in relation to the 50% IC_50_ to perform a second proliferation assay. The WEHI-3 cell line or normal mouse bone marrow mononucleated cells were cultured for 72 and 120 h, respectively, with the IC_25_ and IC_50_ of SC, cytarabine, and daunorubicin, both individually and in combination. After the incubation time, cell proliferation was evaluated by the crystal violet technique, and the data obtained were reported in percentage of proliferation.

#### 2.1.4. Cell Apoptosis Measurement by Annexin V-FITC Assay

Translocation of phosphatidylserine molecules from the inner to the outer layer of the cell membrane was detected with an annexin V-FITC kit (BD Biosciences, San Jose, CA). This phenomenon is representative of early apoptotic stages [20]. Briefly, the cells were washed and incubated with FITC-labelled annexin V for 15 min, and the samples were analyzed by flow cytometry (FACSAria II; BD Biosciences, San Jose, CA).

### 2.2. In Vivo Studies

#### 2.2.1. Animals

Male Balb/c mice between two and three months of age were used and maintained in pathogen-free conditions. The experiments were carried out in the Animal Facility of Zaragoza School of Advanced Studies, National Autonomous University of Mexico, in accordance with the institutional guidelines. The mice were provided with autoclaved water and fed a standard powdered rodent diet *ad libitum*. All experimental protocols were approved with the number FESZ/DEPI/CI/128/14 by the Ethics Committee of Zaragoza Faculty of Advanced Studies, in accordance with the national and international regulations for the care and use of experimental animals.

#### 2.2.2. Establishment of the Leukemia Mouse Model

The WEHI-3 cells were quantified with Trypan blue to confirm >95% viability, washed twice with PBS, and brought to a cell concentration of 1 × 10^6^/mL from PBS. In each test (independent or combined doses), the mice received 2.5 × 10^5^ cells in 250 *μ*L of PBS as vehicle.

#### 2.2.3. Experimental Design and Treatments

The most effective doses of each drug had been previously established [21] and nine experimental groups with *n* = 5 each were constructed as follows:

- Control (control without WEHI-3)
- Control + WEHI-3
- Vehicle (PBS) + WEHI-3
- SC + WEHI-3 (2 mg/kg)
- Cytarabine + WEHI-3 (3 mg/kg)
- Daunorubicin + WEHI-3 (0.5 mg/kg)
- SC : cytarabine (2 mg/kg : 3 mg/kg) +WEHI-3
- SC : daunorubicin (2 mg/kg : 0.5 mg/kg) + WEHI-3
- Cytarabine : daunorubicin (3 mg/kg : 0.5 mg/kg) + WEHI-3

The individual agents or combined treatments were dissolved in 1.0 mL of sterile PBS per mouse. In treatments combined with SC, the antineoplastic agents were dissolved in the sterile solution of SC. One group of mice that served as a control was treated only with PBS (1 mL). All the treatments were inoculated i.p. and started 48 h after cell inoculum and every 48 h thereafter for 35 doses in a period of 70 days. Survival rate was recorded every 24 h and plotted by Kaplan–Meier analysis (SPSS Inc., Chicago, IL, USA).

#### 2.2.4. Leukemic Mouse Bone Marrow Mononuclear Cell Proliferation Assay

On the 30th and 60th days of treatment, one individual from each condition was euthanized, and the femur and the bone marrow cells within it were recovered. BMMNCs were cultured *in vitro* in 96-well plates, in the absence and presence of rmIL-3 as exogenous growth factor, and proliferation was evaluated at 120 h by the crystal violet procedure indicated above.

### 2.3. Statistical Analyses

All individual experiments were carried out in triplicate. All experiments were repeated three times, and the values are expressed graphically as the average values ± SD. One-way Tukey was used for statistical analysis, and *p* < 0.001 was considered statistically significant. Statistical software (SPSS Inc., Chicago, IL, USA) was used to perform the analyses.

## 3. Results

### 3.1. Cell Proliferation Assay

**Figure 1:**
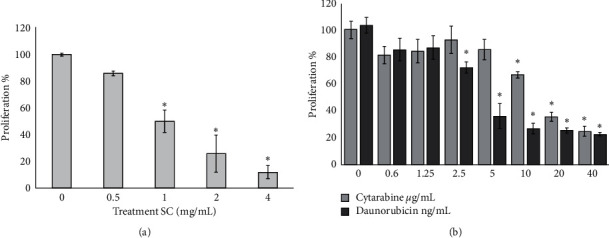
Cell proliferation assay. Cell proliferation curve in WEHI-3 cells treated with different concentrations of sodium caseinate (SC) (a) and cytarabine and daunorubicin (b). The cell proliferation was evaluated by crystal violet technique after 72 h of culture. Values are mean ± SD for three different independent experiments.  ^*∗*^Results are significantly different from control (0) with Tukey test (*p* ≤ 0.001).

To analyze the possible synergistic effect on the inhibition of WEHI-3 proliferation in the presence of SC, cytarabine, or daunorubicin, the cells were subjected to a concentration-dependent proliferation inhibition assay. The results indicate that each of the compounds inhibits proliferation in a concentration-dependent manner with a significant difference from 1 mg/mL for SC (Figure 1(a)), 10 *μ*g/mL for cytarabine, and 2.5 ng/mL for daunorubicin (Figure 1(b)).

### 3.2. The SC Combination with Daunorubicin and Cytarabine Shows the Synergic Effect in the Proliferation Inhibition of WEHI-3 Leukemic Cells and Apoptosis Induction

**Figure 2:**
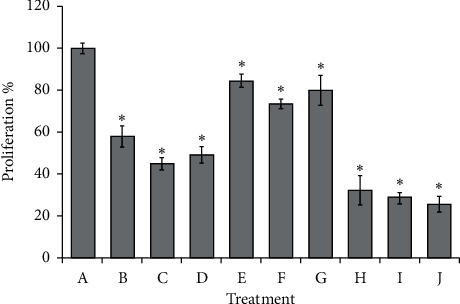
Percentage of proliferation inhibition induced on WEHI-3 leukemia cells with the IC_25_ or IC_50_ of sodium caseinate (SC), cytarabine, or daunorubicin alone or in combination. PBS (A), SC IC_50_ (B), cytarabine IC_50_ (C), daunorubicin IC_50_ (D), SC IC_25_ (E), cytarabine IC_25_ (F), daunorubicin IC_25_ (G), SC : cytarabine IC_25_:IC_25_ (H), SC : daunorubicin IC_25_ : IC_25_ (I), cytarabine : daunorubicin IC_25_ : IC_25_ (J). Values are mean ± SD for three different independent experiments.  ^*∗*^*p* ≤ 0.001 with the Tukey test, group I (B, C, and D column), group II (E, F, and G columns), and group III (H, I, and J columns) are different with respect to control (A column) and different from each other.

Using the data in Figure 1, the IC_50_ calculated for each treatment was 1.85 mg/mL, 17.8 *μ*g/mL, and 5.7 ng/mL of SC, cytarabine, and daunorubicin, respectively. In order to experimentally validate the IC_50_, WEHI-3 cells were cultured in the presence of the respective IC_50_. The results coincide with the reduction of proliferation to the 50% control without treatment. On the other hand, in order to have proliferation inhibition values lower than the IC_50_, the IC_25_ of each compound, we use 0.925 mg/mL, 8.9 *μ*g/mL, and 2.85 ng/mL of SC, cytarabine, and daunorubicin, respectively. The results indicate that, in all cases, there is a proliferation value of around 75% compared to control without treatment (Figure 2). In order to evaluate the combined effect of the compounds, it was found that the IC_25_ combination of SC : cytarabine only reached 30% of proliferation while in the combination SC : daunorubicin and cytarabine : daunorubicin it was less than 25% compared to the control, with significant differences in the values below that reported for the individual IC_50_, even below the total inhibition induced by each of the compounds of the IC_25_ concentration, which highlights the synergistic effect of the combination to reduce proliferation (Figure 2).

To identify the translocation of phosphatidylserine, an early event of apoptosis induction, doses of IC_50_ and IC_25_ of the SC or antineoplastic drugs and the combination of their IC_25_ were analyzed by the annexin V technique. The data reveal that the IC_25_ of each induces early apoptosis pointed out by phosphatidylserine translocation in no more than 16% of the population, close to 9.5% of the control without treatment, but the IC_50_ increases to more than 40% of the annexin V-positive population, while the combination with IC_25_ of SC with cytarabine or daunorubicin more than 74% of the population undergoes phosphatidylserine translocation (Table 1).

### 3.3. The SC or Combination SC-Cytarabine or SC-Daunorubicin Promotes the Proliferation in Bone Marrow-Mononuclear Cells of Normal Balb/c Mice

**Figure 3:**
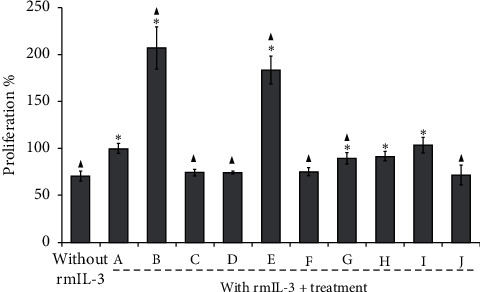
Percentage of proliferation of Balb/c mouse bone marrow mononuclear cells (BMMNCs) in the presence of 5 ng/mL rmIL-3 with or without or IC_25_ or IC_50_ of sodium caseinate (SC), cytarabine, or daunorubicin alone or in combination. PBS (A), SC IC_50_ (B), cytarabine IC_50_ (C), daunorubicin IC_50_ (D), SC IC_25_ (E), cytarabine IC_25_ (F), daunorubicin IC_25_ (G), SC : cytarabine IC_25_ : IC_25_ (H), SC : daunorubicin IC_25_ : IC_25_ (I), cytarabine : daunorubicin IC_25_ : IC_25_ (J). Each value is the mean ± SD of at least three independent assays.  ^*∗*^*p* ≤ 0.001 with the Tukey test with respect to control without rmIL-3; ▲*p* ≤ 0.001 with the Tukey test with respect to 5 ng/mL rmIL-3 + PBS (A).

In order to compare the effect of SC, cytarabine, and daunorubicin on the proliferation of normal cells, culture of BALB/c BMMNC was performed and stimulated with the same doses (IC_25_ and IC_50_), as done in Figure 2, with the individual compounds and in combination. Cells cultured without rmIL-3 did not proliferate, but they did so significantly in their presence of PBS/rmIL-3, while those stimulated with rmIL-3 plus the IC_25_ and IC_50_ of CS increased their proliferation in more than 60% compared to control + PBS/rmIL-3 (Figure 3). Likewise, regarding the addition of IC_25_ or IC_50_ of cytarabine or daunorubicin in BMMNC, despite the presence of rmIL-3, there is no proliferation since it remains at levels of cultures without rmIL-3 (Figure 3). The addition of the combined doses of IC_25_ cytarabine : daunorubicin does not proliferate either, while cell in the presence of the combination SC-cytarabine or SC-daunorubicin proliferates in a similar way to the levels of cell in control PBS/rmIL-3 (Figure 3).

### 3.4. Leukemic Balb/c Mice Treated with SC Combined with Cytarabine or Daunorubicin Have Survivors for More Than 70 Days

**Figure 4:**
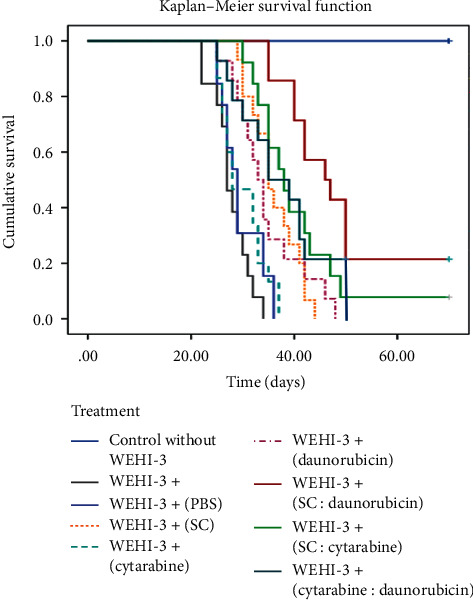
Survival of Balb/c leukemic mice treated with 1 mL of vehicle (PBS) or sodium caseinate (SC) concentration (2 g/kg); cytarabine (3 mg/kg) or daunorubicin (0.5 mg/kg) alone or combined in PBS w/v every 48 h. Healthy mice were included as a control. Kaplan–Meier curve, *n* = 5 per group. PBS: phosphate-buffered saline.

We previously established that the dose of 2 g/kg SC, 3 mg/kg cytarabine, and 0.5 mg/kg for daunorubicin increases the survival of leukemic mice [22]; these concentrations were used and administered via i.p. every 48 h alone or in different combinations (SC : cytarabine, SC : daunorubicin, or cytarabine : daunorubicin), plus a healthy control group in which leukemia was not induced, a WEHI-3 + control without treatment, and a WEHI-3 + control treated with only vehicle. It was found that cytarabine, vehicle, or WEHI-3 + control had a similar survival curve but not more than 38 days; SC or daunorubicin was 44 and 48 days, respectively, while the combinations achieved longer survival; cytarabine : daunorubicin had a survival of 41% at 40 days and zero at 50 days, SC : cytarabine induced a survival of 40% at 40 and remained at 10% for more than 70 days, and finally SC : daunorubicin showed 85% survival at 40 and 20% at 70 days (Figure 4).

### 3.5. BMMNCs Culture from Balb/c Leukemic Mice Shows Proliferation Even without rmIL-3 except Cytarabine : Daunorubicin Treatment

**Figure 5:**
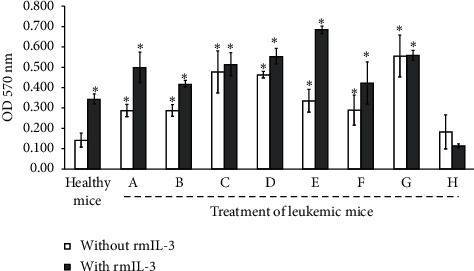
Proliferation of mononucleated cells from bone marrow of leukemic mice at 30 days postinduction of WEHI-3 and treatments, in the absence or presence of rmIL-3. Cultivated for 120 h and evaluated by the crystal violet technique. Control (A), PBS (B), SC (2 g/kg) (C), cytarabine (3 mg/kg) (D), daunorubicin (0.5 mg/kg) (E), SC : cytarabine (F), SC : daunorubicin (G), and cytarabine : daunorubicin (H). Data shown are mean ± SD of three independent experiments,  ^*∗*^*p* ≤ 0.001 with the Tukey test with respect to healthy mice without rmIL-3.

Figure 4 shows that, 30 days after the induction of leukemia, all the treatments had live specimens; thus, mice from each treatment were euthanized to evaluate if the bone marrow had proliferating cells in the absence of rmIL-3 (self-proliferating), a cytokine essential for the growth of mononucleated cells from the bone marrow of healthy mice and which is also constitutively expressed by WEHI-3 cells, such that if proliferation occurs, it indicates the presence of leukemic cells. After 120 h of culture both in the individual treatment groups and in the combined doses, the cells proliferate in the absence of rmIL-3, which reveals the presence of self-proliferating and therefore leukemic cells, except in the cytarabine : daunorubicin treatment where they did not proliferate even with the addition of rmIL-3, which is an indicator of bone marrow damage (Figure 5).

### 3.6. BMMNCs Culture from Balb/c Leukemic Mice Survivor by 60 Days Does Not Show Self-Proliferating Cell

**Figure 6:**
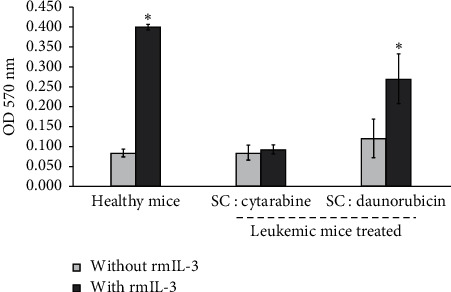
Proliferation of mononucleated cells from bone marrow of leukemic mice at 60 days postinduction of WEHI-3 and treatments, in the absence or presence of rmIL-3. Cultivated for 120 h and evaluated by the crystal violet technique. Data shown are mean ± SD of three independent experiments,  ^*∗*^*p* ≤ 0.001 with the Tukey test with respect to healthy mice without rmIL-3.

Taking into consideration the fact that the SC-cytarabine and SC-daunorubicin treatment promote the survival of leukemic mice at 60 days, we proceeded to assess whether the bone marrow cells still contain self-proliferating cells. It was found that neither of the two surviving groups was observed to have self-proliferating cells; the addition of rmIL-3 promoted proliferation; however, in the treatment of SC-cytarabine, the cells did not proliferate yet with rmIL-3, a sign of bone marrow damage (Figure 6). These results point out that, at 60 days, the treatment with SC-daunorubicin eradicates the autoproliferating cells present at 30 days of treatment and without damage to the BMMNC, which suggests that this combination of compounds eliminates leukemic cells from the bone marrow of leukemic mice.

## 4. Discussion

In this study, we demonstrate that SC combined with cytarabine or daunorubicin has a synergistic antiproliferative effect on WEHI-3 myelomonocytic leukemia cell line compared to the same individual drugs. Here, it is confirmed that SC inhibits proliferation and induces apoptosis in the cell line WEHI-3 (Figure 1 and Table 1), which has been reported in the literature [16]. It is also shown that daunorubicin and cytarabine do the same; in addition, cytarabine has a higher IC_50_ than daunorubicin, which coincides with that reported in the literature [23]. On the other hand, the combination of IC_25_ of SC with daunorubicin or cytarabine enhanced the activity of the two treatments reaching 70% inhibition of proliferation, a value significantly higher than that found in the individual treatments (Figure 2), and more than 74% of WEHI-3 cells undergo phosphatidylserine translocation (Table 1). This demonstrates that combination of SC and daunorubicin or cytarabine with small doses has a strong antileukemic effect.

As previously reported, cytarabine and daunorubicin are toxic, alone or in combination, to hematopoietic cells, especially daunorubicin, which can further damage cardiomyocytes [7, 24]. However, the combinations of these drugs with SC reversed the antiproliferative effect of each antineoplastic, since both combinations have a proliferation similar to culture of the BMMNCs in the presence of rmIL-3 (Figure 3), marking a milestone in the search for molecules with therapeutic potential because SC reduces the damage to bone marrow cells caused by cytarabine and daunorubicin.

These data coincide with the induction of survival in leukemic mice since the most successful treatment was that of SC-daunorubicin where 40% of the mice survived at day 50 and 20% lasted until day 70 (Figure 4), and we have two mice in this group, with more than 6 months of survival. It should be noted that daunorubicin alone or in combination was administered uninterruptedly every 48 h and at half the dose that is used clinically, all in accordance with the fact that the SC promotes survival of leukemic mice [15, 16] and, also, the effectiveness of cytarabine and daunorubicin as antileukemic *in vivo* [25]. We believe that the reduction in the dose of daunorubicin and its combination with SC, which reduces side effects and enhances the antileukemic activity, made this treatment the most successful, since that, in *in vivo* studies of the liposomal drug CPX-351, which is also a combination of drugs that is administered in a constant and controlled-release manner in the body, 100% of the mice survive more than 50 days after the induction of leukemia; however, the WEHI-3 line is used to induce nude CD-1 mice, since the origin of this cell line comes from the mice Balb/c [26]. With the possibility that the active immune system is the one that promotes survival and not the liposomal drug, therefore the results are not completely comparable.

On the other hand, the BMMNCs of the leukemic mice surviving during 30 days of treatment with the combination of cytarabine-daunorubicin did not proliferate even with the addition of rmIL-3; this inability of the BMMNCs to proliferate *in vitro* is an obvious indicator of bone marrow damage induced by treatment with both antineoplastic agents. These data invite us to reflect on the efficacy of cytarabine and daunorubicin as antileukemics *in vivo* [25], especially considering that overall survival is not modified despite the low doses of both compounds contained in Formulation CPX-351 [11].

Additionally, we showed that all BMMNCs from survival mice by 30 day, treated or not with SC, cytarabine, or daunorubicin alone or in combination, proliferated in the absence of rmIL-3 (Figure 5), a cytokine constitutively expressed by WEHI-3 cells [27], for which a proliferant BMMNC culture in the absence of rmIL-3 is indicative of the presence of WEHI-3 cells in the bone marrow. This data confirms the persistence of leukemia in the bone-target to cytarabine or daunorubicin, increasing marrow as we have reported before with SC treatment [16].

After 60 days of treatment, BMMNCs of mice treated with SC-cytarabine were placed in culture and they did not proliferate in the presence of rmIL-3 (Figure 6). This means that there is damage to bone marrow and that, despite prolonged survival of the mice, cytarabine damages hematopoietic cells even in the presence of SC. However, in the culture of the cells of the surviving leukemic mouse treated with SC-daunorubicin, the cells cultured in the presence of rmIL-3 proliferated such as the culture of a healthy mouse. Interestingly, these cells in the absence of rmIL-3 do not have significant proliferation; thus, the elimination of leukemia cells in the bone marrow was confirmed despite being present 30 days after the onset of leukemia (Figure 5). These data are important since we know that the cytarabine and daunorubicin scheme is effective in young patients; however, elderly patients with AML respond poorly to conventional chemotherapy and only selected older adults can tolerate and benefit from standard therapies [28, 29], as a group older adults are more likely to experience treatment-associated toxicity and less likely to benefit from treatment when undergoing standard induction and post-remission therapies [30]. Probability result from differences in the biology of the leukemic blasts in older versus younger patients, or a combination of these factors [29, 31], also has shown that elderly patients that show karyotype anomalies, especially those in the high‐risk category, do not benefit from intensive chemotherapy [32, 33]. Thus, the SC : daunorubicin combination seems to overcome the difficulties of two classic antineoplastics against AML, one of the most common types of leukemia in adults according to the American Society of Clinical Oncology [34].

The mechanism of protection and action of SC : daunorubicin is not known but it is known that an alternative treatment for AML, known as CAG, consists of a low-dose regimen of cytarabine, aclarubicin, and G-CSF [21]. G-CSF influences the bone marrow microenvironment by mobilizing regulatory T cells (Tregs) and myeloid-derived suppressor cells (CSDMs). It also promotes the eviction of leukemic cells from their medullary microenvironment and/or the interruption of signaling; this may explain the increase in cell death observed with cytarabine and anthracyclines since, by mobilizing leukemic cells, leaving them unprotected from BM they are more susceptible to these drugs [35]. We knew that SC promoted granulopoiesis by increasing M-SCF, G-SCF, and GM-SCF [13], and additionally also promoted mobilization of LSK cell mice [14]; thus, it is likely that, by combining SC with cytarabine or daunorubicin, it mobilized the leukemic cells anchored to the hematopoietic niche, taking them into the bloodstream, and therefore accessible to antineoplastic drugs; at the same time, SC allows the proliferation of BMMNC cultured with rmIL-3, which represents their effectiveness and promoting survival.

## 5. Conclusions

It has been published that caseins, the main milk protein, exert a cytotoxic effect in leukemia cell lines, but they promote the proliferation of hematopoietic normal cells, while their administration in leukemic mice promotes survival for more than 40 days, but without eradication of leukemic cells. Here, it is shown that *in vitro* the combination of the IC_25_ of SC-cytarabine or SC-daunorubicin synergizes in the elimination of leukemic cells, with evident induction of death by apoptosis, while the proliferation of mononuclear cells of bone marrow is not affected. *In vivo*, the combined administration of SC-daunorubicin or SC-cytarabine promotes the highest survival rate at 40 days and, in addition, the bone marrow of survivors for more than 60 days, while their BM does not have leukemic cells. The data suggest that the SC-daunorubicin combination may be a compound with possible application as an antileukemic agent.

## Figures and Tables

**Table 1:** Apoptosis induction percentage of WEHI-3 cells treated with the IC_50_ and IC_25_ of SC, cytarabine, and daunorubicin, as well as their combinations, after 72 h of culture and evaluated with the annexin V-FITC and 7AAD technique.

Treatment	Early apoptosis	Necroapoptosis	Necrosis
0	9.49	0	0.03
SC IC_25_	12.15	0.37	10.75
SC IC_50_	64.71	2.04	3.62
Cytarabine IC_25_	9.41	0.37	5.51
Cytarabine IC_50_	46.09	1.54	12.32
Daunorubicin IC_25_	15.72	0.44	4.35
Daunorubicin IC_50_	44.71	1.83	3.98
SC : cytarabine IC_25_ : IC_25_	74.59	3.96	1.56
SC : daunorubicin IC_25_ : IC_25_	73.02	3.01	0.97
Cytarabine : daunorubicin IC_25_ : IC_25_	78.16	4.16	0.88

## Data Availability

The data used to support the findings of this study are included within the article.
